# Comparative transcriptional survey between laser-microdissected cells from laminar abscission zone and petiolar cortical tissue during ethylene-promoted abscission in citrus leaves

**DOI:** 10.1186/1471-2229-9-127

**Published:** 2009-10-23

**Authors:** Javier Agustí, Paz Merelo, Manuel Cercós, Francisco R Tadeo, Manuel Talón

**Affiliations:** 1 Instituto Valenciano de Investigaciones Agrarias - Centro de Genómica. Carretera Moncada-Náquera Km. 4,5. 46113 Moncada (Valencia) Spain; 2 Gregor Mendel Institute of Plant Molecular Biology, Austrian Academy of Sciences, Dr. Bohr-Gasse 3, 1030 Vienna, Austria

## Abstract

**Background:**

Abscission is the cell separation process by which plants are able to shed organs. It has a great impact on the yield of most crop plants. At the same time, the process itself also constitutes an excellent model to study cell separation processes, since it occurs in concrete areas known as abscission zones (AZs) which are composed of a specific cell type. However, molecular approaches are generally hampered by the limited area and cell number constituting the AZ. Therefore, detailed studies at the resolution of cell type are of great relevance in order to accurately describe the process and to identify potential candidate genes for biotechnological applications.

**Results:**

Efficient protocols for the isolation of specific citrus cell types, namely laminar abscission zone (LAZ) and petiolar cortical (Pet) cells based on laser capture microdissection (LCM) and for RNA microextraction and amplification have been developed. A comparative transcriptome analysis between LAZ and Pet from citrus leaf explants subjected to an *in-vitro *24 h ethylene treatment was performed utilising microarray hybridization and analysis. Our analyses of gene functional classes differentially represented in ethylene-treated LAZ revealed an activation program dominated by the expression of genes associated with protein synthesis, protein fate, cell type differentiation, development and transcription. The extensive repertoire of genes associated with cell wall biosynthesis and metabolism strongly suggests that LAZ layers activate both catabolic and anabolic wall modification pathways during the abscission program. In addition, over-representation of particular members of different transcription factor families suggests important roles for these genes in the differentiation of the effective cell separation layer within the many layers contained in the citrus LAZ. Preferential expression of stress-related and defensive genes in Pet reveals that this tissue is likely to be reprogrammed to prevent pathogen attacks and general abiotic stresses after organ shedding.

**Conclusion:**

The LCM-based data generated in this survey represent the most accurate description of the main biological processes and genes involved in organ abscission in citrus. This study provides novel molecular insight into ethylene-promoted leaf abscission and identifies new putative target genes for characterization and manipulation of organ abscission in citrus.

## Background

Abscission of plant organs takes place through a highly coordinated sequence of biochemical events that occur in a discrete group of cells located in predictable positions in the plant, known as abscission zones (AZs) [1]. Shedding of citrus fruits and leaves is regulated by developmental, hormonal and environmental cues [2-5]. In particular, gibberellins [6,7] and carbohydrates [8,9] have been involved in the control of abscission of reproductive organs during the fruit set period. Senescent and aged citrus leaves are shed through the activation of the AZ located at the branch to petiole junction, while stressful environmental conditions such as drought, salinity and subfreezing temperatures stimulate mature leaf abscission at the laminar AZ (LAZ), located at the interface between the petiole and the leaf blade [10-13]. There is evidence supporting the idea that ethylene operates as a hormonal regulator accelerating leaf abscission under many of these adverse environmental conditions [5]. Indeed, ethylene treatments are used to promote fruit loosening in order to facilitate and coordinate mechanical harvesting of citrus fruits [3] although it can also cause excessive leaf abscission and gummosis (a phenomenon by which patches of a gummy substance are formed on the surface of certain plants, particularly fruit trees). In this regard, understanding the regulatory effects of ethylene on abscission is important for the citrus fruit industry.

Enzymatic and gene expression studies on citrus leaf abscission have revealed that the effective separation of cells is a consequence of the increase in activity of several hydrolytic enzymes secreted to the cell walls [14-17]. In a previous analysis of transcriptome changes during ethylene-induced abscission in LAZ-enriched tissues and petioles of debladed leaf explants [18], we described the preferential accumulation of several members of different gene families involved in cell-wall modification, lipid transport, protein biosynthesis and degradation, signal transduction and transcription control pathways in LAZ-enriched tissues after ethylene treatment. However, information about the regulatory signals acting at the onset of the process is rather scarce and mostly limited to the identification of a few transcription factors and protein kinases, and other genes involved in hormonal, calcium and G-protein-related signaling [18-20]. Since these studies were performed on AZ-enriched tissues, the analyzed samples were not ideally homogeneous and invariably included a mixture of cells. Although this approach has been widely used and provided very valuable information, more accurate and promising methods are currently available to investigate the biological processes of the AZ with high precision. Laser capture microdissection (LCM), for instance, may provide invaluable samples of specific cell types for further analyses and proper comparisons [21]. Moreover, the use of LCM followed by transcriptome profiling has proved its potential to identify new candidate genes for abscission control of floral organs in *Arabidopsis *[22].

In this survey, we carried out a high-throughput molecular analysis of the specific gene expression taking place in LAZ and Pet from citrus leaf explants after 24 h of ethylene treatment. Cell-type specific samples were isolated by LCM and amplified mRNA was labelled with either Cy5 or Cy3 and subjected to dye-swap hybridization analysis using a 7K gene citrus microarray [23]. The results notably increase the current catalogue of genes and gene families related to the abscission process, in general, and in citrus, in particular, and provide new candidate genes for biotechnological applications.

## Results and Discussion

### Morphological characterization of ethylene-promoted citrus leaf abscission

**Figure 1:**
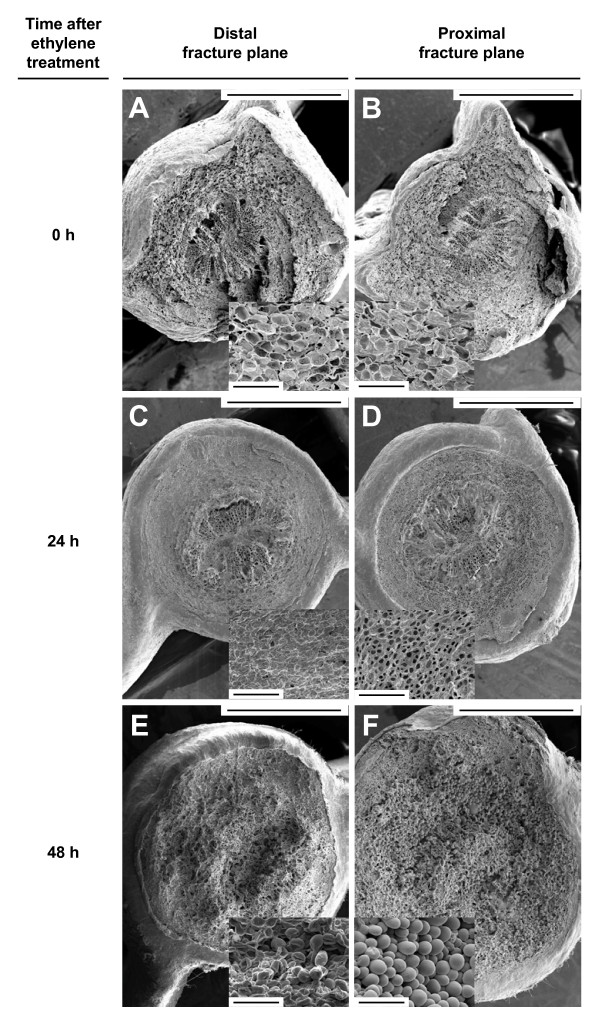
**Cellular morphology of fracture planes at the laminar abscission zone**. Scanning electron micrographs of the distal (A, C and E; leaf-blade side) and the proximal (B, D and F; petiole side) fracture planes of the citrus laminar abscission zone from *Citrus clementina *mature leaf explants non-treated (A and B) and treated for 24 h (C and D) and 48 h (E and F) with ethylene. High magnification pictures show cells of the cortical portion of the laminar abscission zone. Bars: 1 mm and 100 μm.

Scanning electron microscopy was used to examine the cellular morphology of both the distal (leaf-blade side) and the proximal (petiole side) fracture planes of the LAZs from citrus leaf explants at the onset and after 24 and 48 h of ethylene treatment (Figure 1). Before ethylene treatment (Figures 1A, B), both fracture planes showed a ragged surface of broken cell walls, indicating that forcible separation before ethylene promotion of abscission results in the breaking of primary walls due to a high cell adhesion strength in the LAZ. At this stage, abundant plastids were observed inside the LAZ in the distal fracture plane (Figure 1A). A lower force was needed for detachment of the leaf blade from the petiole after 24 h of ethylene treatment (data not shown). Flattened distal and proximal fracture planes were observed at the cortical portions of the LAZ, whereas the vascular cylinder and the pith showed broken cell walls (Figures 1C, D), suggesting that cell separation was activated in the cortex but not yet in the central core of the LAZ. Observation at higher magnification revealed an amorphous material covering the distal fracture plane, whereas the proximal fracture plane showed a smooth surface. The occurrence of this amorphous material may be associated with the accumulation of residual compounds from the partial dissolution of the pectin-rich middle lamella in the cortical portion of the LAZ separation layer, as well as from the dissolution of cell walls. After 48 h of ethylene treatment, the leaf blade fell off at the slightest touch. The cells of both distal and proximal fracture planes showed rounded and elongated cells that seemed to be loosely attached to one another (Figures 1E, F). The micrographs shown in Figure 1 identified samples at three different stages and confirmed previous suggestions that completion of cell separation occurred after 24 h of ethylene treatment [18]. These findings indicate that the abscission program had already started 24 h after ethylene treatment although cell wall loosening and modification was still at an early stage and, therefore, cell separation had not yet occurred. Based on these results, we selected the 24 h ethylene treatment time-point as the ideal one for carrying out transcriptional profiling experiments.

### Laser capture microdissection (LCM) of laminar abscission zone (LAZ) and petiolar cortical cells (Pet) from ethylene-treated citrus leaf explants

**Figure 2:**
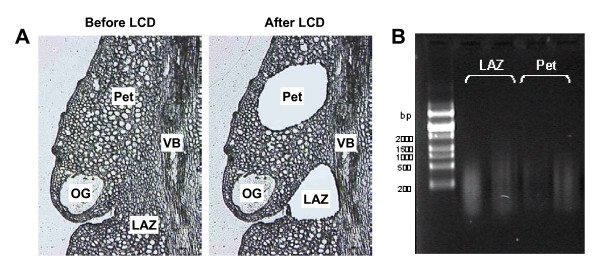
**Anatomy of laminar abscission zone**. Laser microdissected-mediated isolation of laminar abscission zone (LAZ) cells and petiolar cortical (Pet) cells from 10 μm-thick longitudinal sections of *Citrus clementina *mature leaf explants (A). Gel analysis of LAZ and Pet amplified mRNA after two rounds of amplification (B). LAZ = laminar abscission zone; OG = oil gland; Pet = petiolar cortex; VB = vascular bundles.

Abscission has been traditionally studied using hand-dissected AZ-enriched samples that, due to the limited area comprising these zones, are often composed of mixtures of tissues in different proportions. In order to avoid this problem and recover cell-specific samples to perform an accurate study of the abscission events, we took a laser capture microdissection (LCM) approach. We used fresh frozen tissues embedded in OCT medium followed by cryosectioning. This procedure has been reported to produce the best yield of RNA from LCM in animal tissue sources [24,25] as well as in several plant cell types [26-29]. Figure 2A shows that cell morphology in LCM-cells was preserved. LAZ and Pet did not contain ice crystals, a major concern when working with fresh frozen plant tissues.

Laminar AZ and Pet were laser microdissected from citrus leaf explants after 24 h of ethylene treatment (Figure 2A). Microdissected LAZs included cells located in both the adaxial and the abaxial portions of the LAZ. Approximately 15,000 cells were captured per sample and the amount of RNA subsequently isolated per laser-captured cell was approximately 1.5-3 pg. Total RNA recovered from laser microdissected samples was assessed by measurements of OD260/OD280 and then subjected to two rounds of amplification that generated about 80-100 μg of amplified RNA. Gel electrophoresis analysis indicated that the maximum size of aRNA was about 1500 nt (Figure 2B).

### Genes differentially expressed in ethylene-promoted citrus leaf abscission

**Figure 3:**
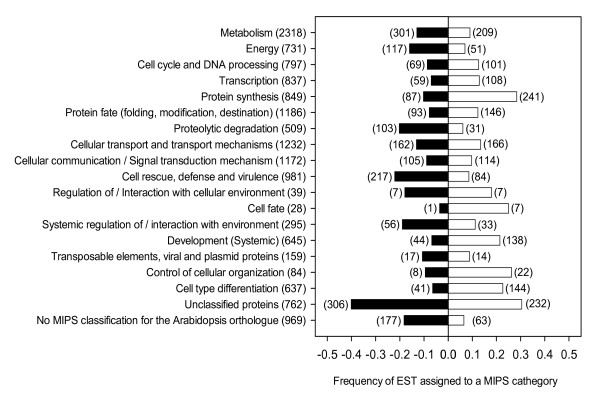
**Distribution of functional cathegories between the laminar abscission zone cells and the petiolar cortical cells**. Ratio and number of ethylene-regulated ESTs in laminar abscission zone cells (open box) or petiolar cortical cells (filled box) of *Citrus clementina *leaves assigned to MIPS (Munich Information Center for Protein Sequences, ) categories. Positive and negative values indicate the EST fraction preferentially expressed in laminar abscission zone cells and petiolar cortical cells, respectively. The total number of ESTs included in each of the MIPS categories is shown in the vertical axis. Data are based on microarray analyses.

Changes in the distribution of gene expression between LAZ and Pet were analyzed 24 h after ethylene treatment using a 7 K unigenes citrus cDNA microarray [23]. Out of 12,672 cDNA microarray probes, 2611 (21%) were differentially expressed between LAZ and Pet. 43% (1133) of them were preferentially expressed in the LAZ whereas 57% (1478) were preferentially expressed in the Pet. All 2611 differentially expressed cDNAs were grouped into functional categories according to the Munich Information Center for Protein Sequences (MIPS; Figure 3). In the LAZ-preferentially-expressed gene set, protein synthesis was the most differentially represented functional class followed by protein fate, cell type differentiation, development and transcription. In the Pet preferentially expressed gene set, cell rescue, defense and virulence, proteolytic degradation and energy were the most prominent functional classes. Notably, the distribution of the cellular communication and transport functional categories was very similar between LAZ and Pet. Other categories involving unclassified proteins and *Arabidopsis *orthologs with no MIPS classification were highly represented both in the LAZ and Pet, suggesting that unknown metabolic processes might be involved in abscission.

The number of ethylene-regulated probes was considerably higher than that previously reported [18] from LAZ-enriched, hand-sectioned tissues and petioles (725 ESTs representing about 6% of the total number of probes). Moreover, MIPS functional classes previously over-represented in petioles, such as protein synthesis, protein fate, cell type differentiation, development and transcription were now preferentially over-represented in LAZ (compare Figure 3 and [18]). This observation clearly established that hand-sectioned LAZ enriched samples were contaminated with significant amounts of non-LAZ tissue, reinforcing the idea that the microdissected analysis and survey are much more accurate and, therefore, that the method is able to determine spatial expression in a more conclusive way. However, MIPS functional classes related to stress response and defense were over-represented in Pet cells in both experiments. These results strongly illustrate the power of LCM to reveal cell-specific distribution of transcripts associated with localized biological processes such as abscission.

The corresponding putative unigenes to all 2611 differentially expressed cDNAs were assigned through the web-browsable database of the Spanish Citrus Functional Genomics Project  in order to identify genes putatively involved in molecular and cellular mechanisms responsible for the regulation of the citrus leaf abscission rate by ethylene.

### Degradation and biosynthesis of cell wall polysaccharides

**Figure 4:**
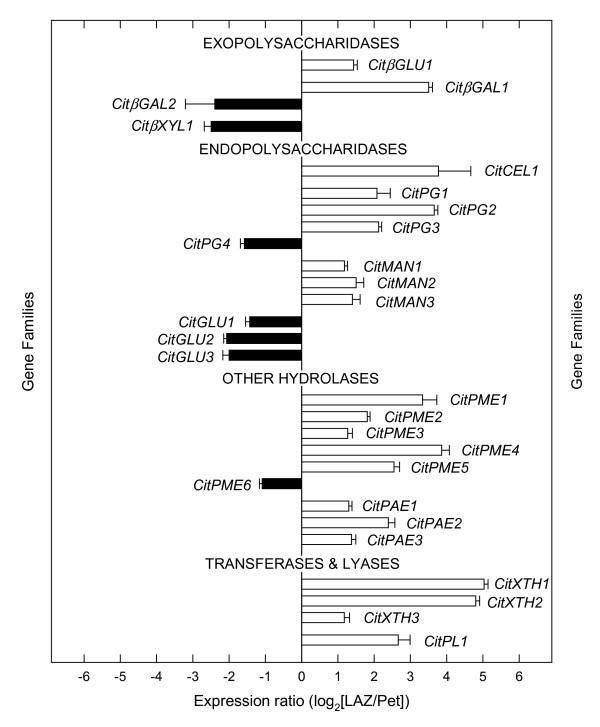
**Expression of genes encoding cell-wall modifying enzymes**. Expression ratio (log_2_) between laminar abscission zone cells and petiolar cortical cells (LAZ/Pet) of genes encoding cell-wall modifying enzymes (exopolysaccharidases, endopolysaccharidases, other hydrolases and transferases and lyases) with significant changes after 24 h of ethylene treatment to *Citrus clementina *leaf explants based on microarray analyses. Positive values show transcripts preferentially expressed in LAZ and negative values those preferentially expressed in Pet. Each bar represents the expression ratio of a singleton or of different ESTs assembled in the same contig. Data are the average of two dye-swap comparisons and error bars show SE.

**Figure 5:**
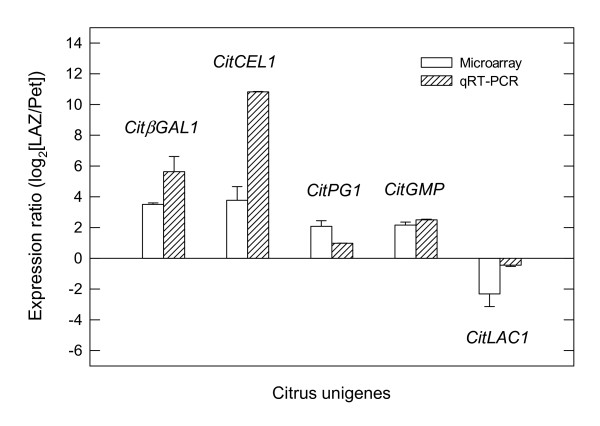
**qRT-PCR analysis of genes related to cell-wall modification**. Expression ratio (log_2_) between laminar abscission zone cells and petiolar cortical cells (LAZ/Pet) of *CitίGAL1*, *CitCEL1*, *CitPG1*, *CitGMP*, *CitLAC1 *based on microarray results and quantitative real-time PCR. The qRT-PCR results confirm the tendency of expression observed in the microarray data.

A large number of ESTs corresponding to genes encoding cell wall hydrolases, transferases and lyases were found to be over-represented in the LAZ 24 h after ethylene treatment (Figure 4; see Additional File 1). Two exopolysaccharidases (a β-glucosidase, *CitβGLU1 *and a β-galactosidase, *CitβGAL1*), seven endopolysaccharidases (an acidic cellulase, *CitCEL1*, three polygalacturonases, *CitPG1*-*3*, and three mannan endohydrolases, *CitMAN1*-*3*), three xyloglucan endotransglucosylases (*CitXTH1*-*3*), a pectate-lyase (*CitPL1*), eight genes encoding other cell wall hydrolases (five pectin-methylesterases, *CitPME1*-*5*, and three pectin-acetylesterases, *CitPAE1*-*3*), as well as a gene encoding a putative expansin (*CitEXP1*) were preferentially expressed in the LAZ (Figure 4). In the Pet, two exopolysaccharidases (a β-galactosidase, *CitβGAL2*, and a β-xylosidase, *CitβXYL1*), four endopolysaccharidases (a polygalacturonase, *CitPG4*, and three β-1,3-glucanases, *CitGLU1*-*3*) and a pectin-methylesterase (*CitPME6*) were preferentially expressed after 24 h of ethylene treatment (Figures 4 and 5).

Abscission of citrus leaves and fruits has been previously associated with increases in the activity of two types of hydrolytic enzymes secreted to the cell walls, namely endo-1,4-β-glucanases (cellulases) and polygalacturonases. Accordingly, the expression of the genes encoding these enzymes as well as others encoding two additional hydrolases, pectin-methylesterases and β-galactosidases have also been reported [15-18,30]. In abscission-activated calyx AZs from *Citrus sinensis *fruit, two different cellulase genes (acidic cellulase *CEL-a1*, and basic cellulase *CEL-b1*) as well as two genes encoding polygalacturonases (*PGI *and *PGIII*) have been isolated [15,31]. Our results show that *CitCEL1*, the homologous gene to *CEL-a1 *in *Citrus clementina*, displays a preferential expression in the LAZ (Figures 4 and 5). Interestingly, none of the three polygalacturonase transcripts over-represented in LAZ cells (*CitPG1*-*3*) showed homology to the previously described *PGI *and *PGIII *genes, thus representing new PGs putatively involved in citrus abscission.

A pectin-methylesterase (*CsPME3*) and a β-galactosidase (*CsβGAL*) have been reported to be up-regulated in *Citrus sinensis *ethylene-activated AZs [16,17]. In our *Citrus clementina *survey, *CitPME6*, the homolog of *CsPME1 *a gene apparently not involved in abscission [16], was preferentially expressed in the Pet, while interestingly, *CitβGAL2*, the homolog of *CsβGAL*, was over-represented not in the LAZ but rather in the Pet (Additional File 1; Figs 4 and 5). Indeed, up-regulation of a β-1,3-glucanase in calyx AZs of *Citrus sinensis *fruit treated with ethylene has also been previously reported [31], while our results indicated that not one but three β-1,3-glucanases (*CitGLU1*-*3*) were also over-represented in the Pet. Again, these unexpected findings may be related to the accuracy achieved with the LCM harvesting in comparison to the traditional manual harvesting. Proteins with β-1,3-glucanase activity are group 2 pathogenesis-related proteins (PR2) involved in limiting pathogen activity, growth and spread in the plant [32]. Therefore, we speculate that CitGLUs, in association with other PRs, could play an important role in the defense program launched by ethylene in Pet during abscission.

Our previous results revealed that in ethylene-treated citrus leaf explants, a pectate-lyase and two xyloglucan endotransglucosylases were over-represented in LAZ-enriched tissues [18]. With the transcriptional survey presented here, we have shown that members of other gene families related to cell wall modification such as β-glucosidases (*CitβGLU1*), mannan endohydrolases (*CitMAN1*-*3*), pectin-acetylesterases (*CitPAE1*-*3*) and expansins (*CitEXP1*) were preferentially expressed in LAZ (Figure 4), thus expanding the list of abscission players putatively involved in cell wall degradation events taking place during citrus leaf abscission.

In addition to cell wall degradation, cell elongation was also observed during the last step of ethylene-treatment along both LAZ fracture planes (Figure 1). This observation correlates with the over-representation of transcripts encoding proteins involved in different metabolic pathways associated with cell wall biosynthesis and cell elongation in the LAZ [33,34] (see Additional File 2). Indeed, a large number of ESTs corresponding to genes involved in purine and pyrimidine metabolism, pyruvate metabolism, glycolysis and nucleotide-sugar interconversions were preferentially expressed in the LAZ after 24 h of ethylene treatment. Expression of these genes might also be connected to the over-representation of four genes encoding 14-3-3 proteins that interact with a wide array of enzymes involved in primary biosynthetic and energy metabolism in plants regulating their catalytic activity [35]. Interestingly, *Citrus *orthologs of several genes encoding 14-3-3-interacting proteins were also preferentially expressed in the LAZ, suggesting a putative link between the expression of the 14-3-3 genes and those related to pyruvate metabolism and glycolysis.

On the other hand, a glycosyltransferase (*CitQUA1*) and a methyltransferase (*CitQUA2*) with high homology to two proteins related to pectin biosynthesis [36,37] and four cellulose synthases (*CitCeS1*-*4*) were preferentially expressed in the LAZ whereas two callose synthases (*CitCaS1 *and *2*) were preferentially expressed in the Pet (see Additional Files 2 and 3). Callose deposition at the proximal side of the LAZ has been observed in senescing leaves of *Citrus sinensis *although its role in abscission is uncertain [38]. In addition, callose plays an important role in plant defense against pathogen attacks [39]. We suggest that its deposition in the Pet could be related to petiole protection after organ shedding.

### Protein biosynthesis and metabolism

A large number of ESTs corresponding to genes encoding ribosomal proteins were over-represented in the LAZ 24 h after ethylene treatment (see Additional File 4). Eighty genes encoding proteins of both ribosomal subunits were preferentially expressed in the LAZ, whereas only four of these genes were preferentially expressed in the Pet. In addition, twice as many genes encoding transcription initiation and elongation factors were preferentially expressed in the LAZ than in the Pet (see Additional File 4). This is in agreement with previous reports showing increases in the surface area of the rough endoplasmic reticulum in the activated calyx AZ of young fruits of lemon, in the LAZ of orange leaves [40,41] and in ethylene-activated AZs of other plant species [42,43]. Thus, our observations suggest that protein synthesis is enhanced in the AZ during ethylene-promoted abscission.

**Figure 6:**
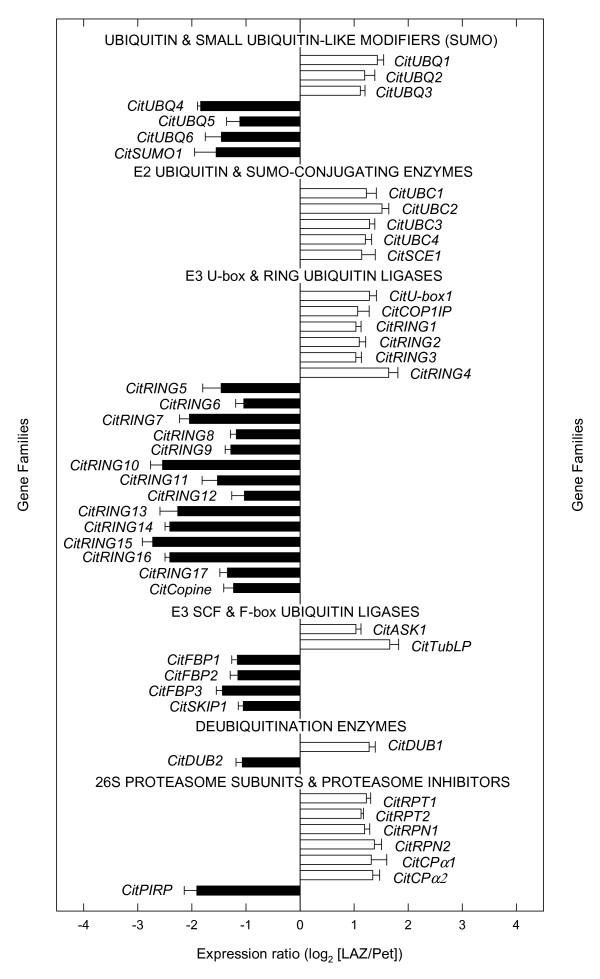
**Expression of genes encoding components of the ubiquitin/proteasome system**. Expression ratio (log_2_) between laminar abscission zone cells and petiolar cortical cells (LAZ/Pet) of genes encoding components of the ubiquitin/proteasome system with significant changes after 24 h of ethylene treatment to *Citrus clementina *leaf explants based on microarray analyses. Positive values show transcripts preferentially expressed in LAZ and negative values those preferentially expressed in the Pet. Each bar represents the expression ratio of a singleton or of different ESTs assembled in the same contig. Data are the average of two dye-swap comparisons and error bars show SE.

The ubiquitin/proteasome system (UPS) has been involved in the signal transduction of developmental and environmental stimuli and in the perception and signaling of plant hormones including ethylene [44]. ESTs corresponding to three genes encoding ubiquitin (*CitUBQ1*-*3*) were preferentially expressed in the LAZ, whereas other UBQs (*CitUBQ4*-*6*) and a SUMO protein (*CitSUMO1*) were preferentially expressed in the Pet (Figure 6), suggesting that UPS is activated by ethylene in both cell types. Interestingly, four E2 ubiquitin-conjugating enzymes (*CitUBC1*-*4*) and a SUMO-conjugating enzyme (*CitSCE1*) were preferentially expressed in the LAZ (Figure 6). In addition, a large number of ESTs corresponding to E3 ubiquitin-ligase genes were also over-represented in both the LAZ and the Pet (Figure 6). These E3 genes were distributed as follows: four RING-finger domain proteins (*CitRING1*-*4*), a U-box domain-containing protein (*CitU-box*), a COP1-interacting protein (*CitCOP1IP*) and two F-box proteins (*CitASK1 *and *CitTubLP*) were up-regulated in the LAZ, whereas thirteen RING-finger domain proteins (*CitRING5*-*17*), a copine-like protein (*CitCopine*) and four F-box proteins (*CitFBP1*-*3 *and *CitSKIP1*) were expressed in the Pet (Figure 6). One of the RING-finger domain genes over-represented in the Pet cells, *CitRING5*, shows a high homology to a RING-H2 finger gene identified in the citrus rootstock *Poncirus trifoliata*, reported to be induced by drought stress and cold [45]. Moreover, additional proteasome components were over-represented in the LAZ in comparison with the Pet (Figure 6), while only a proteasome inhibitor (*CitPIRP*) was preferentially expressed in the Pet. The transcript distribution of the UPS components between the LAZ and the Pet might be of importance since current lines of evidence suggest that this system may play a role in abscission. First, *Arabidopsis *mutants reported to show delayed [46] or arrested [47] floral organ abscission are knock-outs of F-box proteins. Second, in the assembly of 54,000 Citrus ESTs from all plant tissues under different conditions performed by Terol *et al. *[48], an E2 ubiquitin-conjugating enzyme and two E3 ubiquitin-ligases were found to be present exclusively in the abscission-related libraries. Taken together, these observations strongly point to a general proteasome-related mechanism perhaps playing a role in abscission and that certain members of the UPS appear to participate in ethylene-induced abscission.

The UPS has also been recently shown to be involved in plant defense mechanisms mediated by R-proteins [49]. In citrus leaf explants, a small number of transcripts showing homology to UPS components were over-represented in petioles during ethylene treatment and leaf abscission [18]. The UPS components that, in our experimental system, appear to be specifically activated in the Pet during ethylene-induced abscission, could contribute to the activation of defense mechanisms in the tissues that remain attached to the plant as previously suggested [18].

### Defense and interaction with the environment

**Figure 7:**
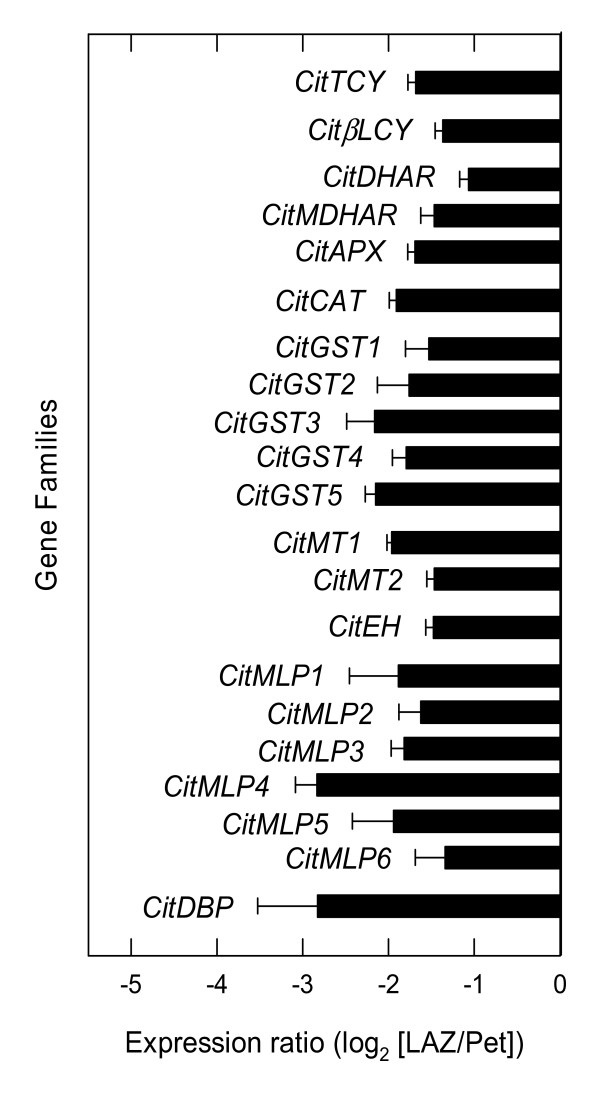
**Expression of genes encoding stress-related proteins**. Expression ratio (log_2_) between laminar abscission zone cells and petiolar cortical cells (LAZ/Pet) of genes encoding stress-related proteins with significant changes after 24 h of ethylene treatment to *Citrus clementina *leaf explants based on microarray analyses. Positive values show transcripts preferentially expressed in LAZ and negative values those preferentially expressed in the Pet. Each bar represents the expression ratio of a singleton or of different ESTs assembled in the same contig. Data are the average of two dye-swap comparisons and error bars show SE.

There is increasing evidence suggesting that reactive oxygen species (ROS) might be associated with ethylene-induced abscission [18,50-52] as well as with other physiological processes that can indirectly provoke organ abscission, such as pathogen attack and senescence [53,54]. In ethylene-treated citrus leaf explants, a set of transcripts belonging to the oxidative stress scavenging machinery (a catalase, a glutathione dehydrogenase, an ascorbate peroxidase and two peroxidases) have previously been reported to be over-represented in petioles whereas a peroxidase was transiently over-represented in manually-dissected LAZ-enriched tissues [18]. Recently, hydrogen peroxide (H_2_O_2_) has been shown to be directly involved in ethylene-mediated abscission signaling *in vitro *in *Capsicum *leaves, where it appears to act as an intermediate molecule in the expression of ethylene-induced cell wall hydrolases [55]. To minimize the damaging effects of ROS, plants have evolved non-enzymatic and enzymatic antioxidant defenses. Non-enzymatic defenses include compounds with intrinsic antioxidant properties, such as vitamins C (ascorbate) and E (α-tocopherol), glutathione and β-carotene. Our data reveal that genes for a tocopherol cyclase and a β-lycopene cyclase involved in the synthesis of vitamin E and β-carotene, respectively, were over-represented in the Pet after ethylene treatment (Figure 7 and Additional File 5). The enzymatic defenses include catalases, peroxidases, superoxide dismutases, the enzymes of the ascorbate-glutathione cycle, metallothionein-like proteins, and glutathione S-transferases (GST). A catalase, *CitCAT*, two metallothionein-like proteins (*CitMT1 *and *2*) and four *GSTs *(*CitGST1-4*) were also over-represented in the Pet after ethylene treatment (Figure 7). Plant metallothionein-like proteins are supposed to be involved in metal ion metabolism or detoxification and citrus metallothioneins have been reported to be highly abundant in developing fruit [56]. The ascorbate-glutathione cycle is operative in chloroplasts and plant mitochondria in order to remove H_2_O_2 _generated during energy metabolism. The cycle is catalyzed by a set of four enzymes, ascorbate peroxidase (APX), monodehydroascorbate reductase (MDHAR), glutathione-dependent dehydroascorbate reductase (DHAR) and glutathione reductase (GR). Interestingly, three of them (*CitDHAR*, *CitMDHAR*, *CitAPX*), were over-represented in the Pet (Figure 7). These results show that ethylene treatment favours the expression of antioxidant genes in the non-abscising tissue adjacent to the LAZ. In citrus plants, epoxide hydrolase and miraculin-like protein (MLPs) genes have been involved in defensive functions against pathogens [57,58]. In this work, an epoxide hydrolase gene (*CitEH*) highly homologous at the amino acid level to RlemEH [57] and six MLP genes (*CitMLP1-6*) were over-represented in the LAZ (Figure 7). In addition, a DNA-binding protein (*CitDBP*) was also over-represented in the LAZ.

**Figure 8:**
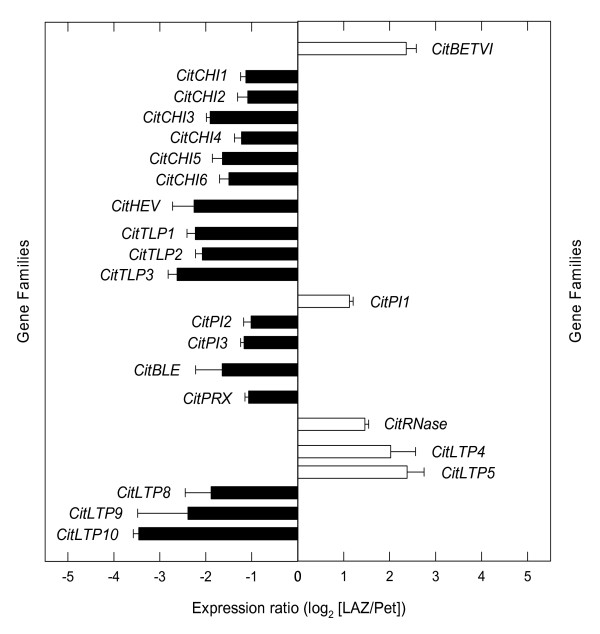
**Expression of genes encoding pathogenesis-related proteins**. Expression ratio (log_2_) between laminar abscission zone cells and petiolar cortical cells (LAZ/Pet) of genes encoding pathogenesis-related proteins with significant changes after 24 h of ethylene treatment to *Citrus clementina *leaf explants based on microarray analyses. Positive values show transcripts preferentially expressed in LAZ and negative values those preferentially expressed in the Pet. Each bar represents the expression ratio of a singleton or of different ESTs assembled in the same contig. Data are the average of two dye-swap comparisons and error bars show SE.

Plant organs respond to biotic and/or abiotic stress accumulating pathogenesis-related proteins (PR proteins) that are grouped into 17 families [32]. There are at least ten PR families (PR-1 to 5, PR-8, and PR-11 to 14 families) whose members have direct activities against fungal pathogens. Hydrolytic activity on cell walls (β-1,3-glucanase) has been demonstrated for members of the PR-2 family and three genes putatively included in this family in *Citrus *(*CitGLU1-3*) were preferentially expressed in the Pet (see Figure 4). Members of the PR-3 and PR-4 protein families are endochitinases (class I and II) and Barwin-like endoglucanases which can hydrolyze chitin from fungal cell walls. Six genes encoding chitinases (*CitCHI1 *to *6*) and a hevein-like endoglucanase (*CitHEV*) were also over-represented in the Pet after ethylene treatment (Figure 8 and Additional File 5). Members of the PR-5 protein family (thaumatin-like proteins) have been related to membrane permeabilization, glucan hydrolysis and apoptosis [32,50-61]. Three thaumatin-like protein genes (*CitTLP1-3*) were preferentially over-represented in the Pet (Figure 8). Proteinase inhibitors (PI), ascribed to the PR-6 family, participate in the response to nematodes and herbivorous insect attacks [62]. A putative PI gene (*CitPI1*) was preferentially expressed in the LAZ, whereas another two (*CitPI2 *and *3*) were over-represented in the Pet. Members of the PR-7 and PR-9 protein families, *CitBLE *and *CitPRX*, respectively, were preferentially expressed in the Pet, whereas a member of the PR-10 protein family (*CitRNase*) was preferentially expressed in the LAZ. Members of the PR-14 protein family are lipid-transfer proteins supposedly involved in membrane permeabilization with antifungal and antibacterial activities [63-65], and three putative LTP genes (CitLTP8 to 10) with homology to protease inhibitor/seed storage/lipid transfer protein proteins were preferentially expressed in the Pet (Figure 8).

In this survey, we have detected that putative members of eight PR gene families were preferentially expressed in the Pet, strongly suggesting that cell separation events taking place in the LAZ might be coordinated with plant defense responses in Pet cells, both triggered by ethylene during the leaf abscission process.

In addition to these defense pathways over-represented in the Pet, six genes encoding nucleotide binding site-leucine rich repeat-containing proteins (NBS-LRRs) were also predominantly expressed in this tissue (see Additional File 5). In general, NBS-LRR-encoding genes are functional in disease resistance and plant defense responses [66,67]. These additional data reinforced the view that ethylene induced the expression of genes encoding defensive proteins in the Pet of citrus leaves to protect tissues remaining attached to the plant from pathogen attack.

### Protein phosphorylation

**Figure 9:**
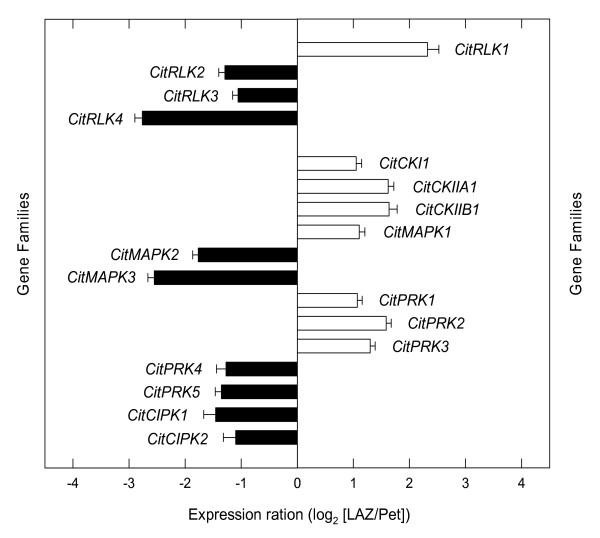
**Expression of genes encoding protein kinases**. Expression ratio (log_2_) between laminar abscission zone cells and petiolar cortical cells (LAZ/Pet) of genes encoding protein kinases with significant changes after 24 h of ethylene treatment to *Citrus clementina *leaf explants based on microarray analyses. Positive values show transcripts preferentially expressed in LAZ and negative values those preferentially expressed in Pet. Each bar represents the expression ratio of a singleton or of different ESTs assembled in the same contig. Data are the average of two dye-swap comparisons and error bars show SE.

Protein phosphorylation and dephosphorylation represent a major form of reversible post-translational modification that controls many regulatory circuits in eukaryotes by modulating the conformation, activity, localization and stability of substrate proteins [68]. In our survey, the expression of three casein kinase genes (*CitCKI*, *CitCKIIA1 *and *CitCKIIA2*), a leucine-rich repeat receptor-like protein kinase (*CitRLK1*), a MAP kinase (*CitMAPK1*) and three serine/threonine protein kinases (*CitPRK1 *to *3*) was favoured in the LAZ after 24 h of ethylene treatment (Figure 9 and Additional File 6). In the Pet, expression of three leucine-rich repeat receptor-like protein kinases (*CitRLK2 *to *4*), two serine/threonine protein kinases (*CitPRK4 *and *5*), two MAP kinases (*CitMAPK2 *and *3*) and two CBL-interacting protein kinases (*CitCIPK1 *and *2*) was preferentially represented (Figure 9).

Different protein kinases have previously been detected in citrus AZs during stress and hormonal-induced abscission. In manually-dissected LAZ-enriched tissues of Clementine mandarin, we recently reported over-expression of two protein kinases, a mitogen-activated protein kinase kinase with homology to AtMKK4 and a serine/threonine protein kinase, between 6-12 h after ethylene treatment [18]. In this species, plants subjected to a water stress/rehydration cycle showed preferential expression of two CDPKs in LAZ-enriched tissues at the onset of rehydration and leaf abscission [20]. Moreover, two partial cDNA sequences showing a peak of expression 8 h after ethylene treatment and with high homology to MAP kinases (*pk41 *and *pk42*) were cloned in mature fruit AZs of *Citrus sinensis *[69]. Applying a strategy of subtractive cDNA library screening, Burns [3] reported the isolation of three protein kinases from the calyx AZ of mature fruit treated with the abscission agent 5-chloro-3-methyl-4-nitro-1H-pyrazole. Increased expression of six protein kinase genes in the stamen AZ was also detected under natural stamen abscission in *Arabidopsi*s flowers [22]. In *Arabidopsis*, abscission of floral organs is delayed in plants with suppressed expression of the receptor-like kinase HAESA/RLK5 [70]. Reporter gene expression, RT-PCR, and in situ hybridization analysis pointed out that three RLKs (RLK1, RLK7 and RLK902) are highly expressed in *Arabidopsis *floral organ AZs [71,72]. The final step of floral organ abscission in *Arabidopsis *is controlled by INFLORESCENCE DEFICIENT IN ABSCISSION (IDA) acting as a ligand interacting with two receptor-like kinases (HAESA, HAE and HAE-like 2, HSL2) [73]. A recent genetic study demonstrated that a signaling cascade from ligands (IDA) to receptors (HAE, and HSL2) to cytoplasmic effectors (mitogen-activated protein kinase kinases, MKK4 and MKK5) function together to control cell separation during abscission [74]. Thus, protein phosphorylation by protein kinases appears to be an active process taking place in AZs during organ separation.

Casein kinases have been shown to be involved in the phosphorylation of particular members of bZIP family proteins necessary to induce downstream gene expression in response to abiotic stress conditions [75,76]. In this regard, it is worth mentioning that the activity of the proteins encoded by the three casein kinases over-represented in the LAZ might be related to that of the LAZ-preferentially expressed CitbZIP1 (Figure 9).

Receptor-like kinases (RLKs) in plants participate in a broad range of developmental processes, including hormone perception, pathogen attack, organ senescence and abscission. CitRLK1 showed high homology to the RLK ERECTA that regulates organ shape and inflorescence architecture in *Arabidopsis *[77] (see Additional File 6). Genetic studies showed that ERECTA and the homeobox gene KNAT1 interact in the regulation of pedicel architecture [78]. In a recent report, KNAT1 has been involved in meristem development and leaf morphogenesis, affecting floral organ AZ development [79]. The above observations strongly suggest a role for ERECTA or an ERECTA-LIKE gene in citrus abscission.

In addition to the differential expression of the protein kinases described above, six protein phosphatases and genes encoding other signaling proteins were over-represented in the LAZ or Pet 24 h after ethylene treatment (Additional File 6).

The results in this section indicate the occurrence of different signaling cascades in either the LAZ or Pet in response to ethylene. We suggest, based on sequence homologies, that some of these genes may be involved in developmental events aimed at the growth of a protective cell layer, conferring a transitory meristematic activity to the AZ.

### Transcription factors

**Figure 10:**
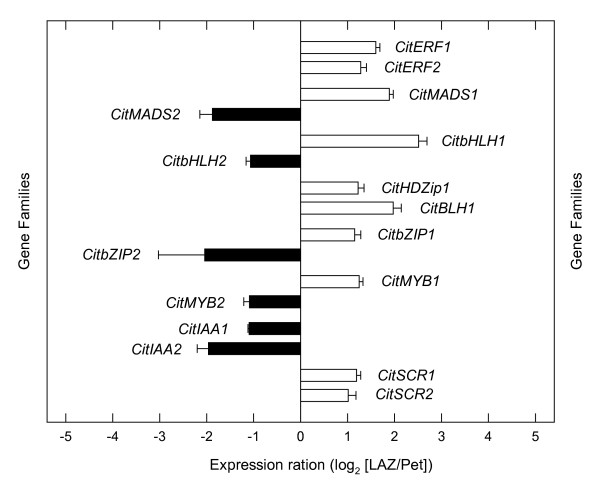
**Expression of genes encoding transcription factors**. Expression ratio (log_2_) between laminar abscission zone cells and petiolar cortical cells (LAZ/Pet) of genes encoding transcription factors with significant changes after 24 h of ethylene treatment to *Citrus clementina *leaf explants based on microarray analyses. Positive values show transcripts preferentially expressed in LAZ and negative values those preferentially expressed in Pet. Each bar represents the expression ratio of a singleton or of different ESTs assembled in the same contig. Data are the average of two dye-swap comparisons and error bars show SE.

**Figure 11:**
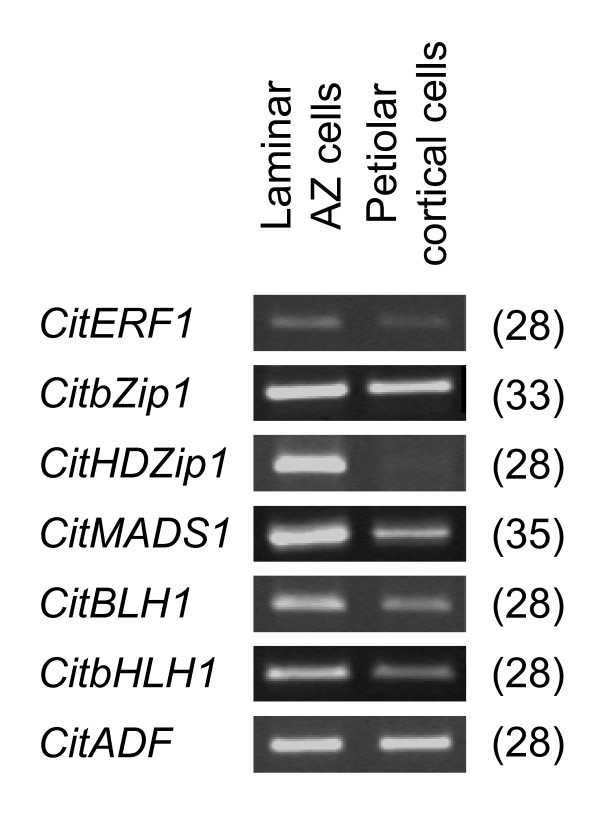
**sqRT-PCR analysis of transcription factors**. Semi-quantitative RT-PCR-based differential expression analyses of *CitERF1*, *CitZip1*, *CitHDZip1*, *CitMADS1*, *CitBLH1*, *CitbHLH1 *and *CitABP *between laminar abscission zone (LAZ) and petiolar cortical (Pet) cells. Numbers on the right hand side represent the cycles performed during the PCR.

A remarkably large number of gene transcripts belonging to different families of transcription factors preferentially accumulated in both the LAZ and the Pet after 24 h of ethylene treatment (Additional File 7). Ethylene favoured the preferential expression of two AP2/ethylene response factors, (*CitERF1 *and *CitERF2*), a MADS-box transcription factor (*CitMADS1*), a basic helix-loop-helix transcription factor (*CitbHLH1*), two homeodomain proteins (*CitHDZip1 *and *CitBLH1*), a basic-region leucine zipper transcription factor (*CitbZIP1*), a MYB transcription factor (*CitMYB1*) and two scarecrow-like proteins (*CitSCR1 *and *CitSCR2*) in the LAZ (Figures 10 and 11). Transcripts encoding other members of some of these families as well as two genes encoding AUX/IAA proteins (*CitIAA1 *and *CitIAA2*) were preferentially expressed in the Pet (Figure 10). This last observation is in agreement with the accepted model of hormonal interactions during abscission that suggests that auxin levels in the AZs prevent ethylene-induced abscission activation (see [[80,81] and [82]] for a review). In the citrus leaf explants used for our survey, the leaf blade was almost completely removed prior to ethylene treatment, thus preventing the supply of auxin to the LAZ.

In general, the transcription factors preferentially expressed in the ethylene-stimulated LAZ display homology to a number of transcription factors previously described in different plant species and involved in the regulation of cell separation processes such as anther and fruit dehiscence or floral organ abscission. Thus, CitERF2 showed a high homology to the *Arabidopsis *ethylene response factor ERF113 (Additional File 7), which has recently been associated with stamen abscission in *Arabidopsis *together with other members of the ethylene response factor gene family [22]. Moreover, several MYB transcription factors isolated from rice and *Arabidopsis*, and implicated in the differentiation of the stomium and the septum, two anther cell types involved in the opening of the stomium and pollen release, have also been related to anther dehiscence [83-85]. Our survey revealed two scarecrow-like proteins (*CitSCR1 *and *CitSCR2*) over-represented in the citrus LAZ. This finding is somehow related to the expression in rice of a scarecrow-like gene, *OsSCR*, in the L1 layer of the ligule primordium [86]. This is exciting since in cereals, the laminar joint at the blade-sheath boundary that carries a pair of auricles and the ligules is localized in a tissue region anatomically equivalent to the LAZ in the citrus leaf. It has also been shown that the gene *liguleless2*, responsible for the definition of a clear blade-sheath boundary in maize, encodes a basic leucine zipper transcription factor [87]. In addition, three genes belonging to the same family have been isolated in ethylene-induced leaf AZs in bean [88]. These TGA-type basic leucine zippers are able to recognize and bind to the ACGT motif in the bean abscission cellulase promoter and are probably involved in the regulation of cellulase expression during leaf abscission. Interestingly, *CitHDZip*, a gene that was up-regulated in our survey for the citrus LAZs shows a high homology to the bean HDZ1 homeodomain-basic leucine zipper protein (Additional File 7), and a potential role in binding the cellulose promoter is suggested.

Our results also revealed up-regulation in the LAZ of a MADS-box gene (*CitMADS1*) that shows high homology at the amino acid level to the *Arabidopsis *SHATTERPROOF1 protein (Additional File 7). This gene is critical for the differentiation of the dehiscence zone of the *Arabidopsis *fruit that is achieved through the coordinated activity of this and several other proteins belonging to the MADS-box (FRUITFULL), basic helix-loop-helix (INDEHISCENT and ALCATRAZ) and homeodomain (REPLUMLESS) transcription factor families (for a review, see [89]). Moreover, CitMADS1 is the Clementine ortholog of the Satsuma mandarin MADS-box CitMADS6 recently isolated from fruit tissue [90]. Another over-represented gene, the putative homeobox protein, CitBLH1, showed high homology to the TALE homeodomain protein, JuBel1, of barley (Additional File 7). It has been shown that JuBel proteins heterodimerized with proteins encoded by class I and II Knox genes [91]. Furthermore, the *Arabidopsis KNAT1 *gene, a class I KNOX homeobox gene, has been proposed to participate in the regulation of abscission zone development [79]. Thus, CitBLH1 might interact with class I KNOX proteins participating in certain abscission events in the LAZ of citrus leaves. In addition to these transcription factors, CitbHLH encoded a basic helix-loop-helix transcription factor highly homologous to the *Arabidopsis *BIGPETAL protein, involved in petal cell expansion [92].

It is worth mentioning that this survey has identified several citrus genes such as *CitMADS1*, *CitBLH1 *and *CitbHLH1*, encoding putative transcription factors that might be associated with cell differentiation and separation events and that were preferentially expressed in the LAZ (Figures 10 and 11).

Thus, the high homology of the preferentially expressed transcription factors in the citrus LAZ with several proteins either involved in cell differentiation or in organ shedding indicates the occurrence of two major groups of transcription factors among the identified genes. The LAZ comprises 15-20 cell layers in citrus [18] and, as a cell type, is differentiated early during leaf development. However, the actual effective separation layer within the abscission zone is not predetermined and apparently is only defined just before the onset of abscission. Therefore, we propose that one of these transcription factor groups operates as a putative regulatory network during the definition of the effective separation layer while the second one may act as an activation controller of citrus leaf abscission.

## Conclusion

Among the cell separation processes, abscission represents a challenge in terms of the experimental approach due to the restricted area in which it takes place. In this work, we developed an accurate protocol for sample preparation and LCM to isolate specific cell types from contiguous tissues involved in ethylene-promoted citrus leaf abscission.

The catalogue of genes associated with cell wall biosynthesis and metabolism preferentially expressed in LAZ during ethylene-induced abscission treatment potentially involved in organ shedding has, thus, been significantly enlarged. The results suggest that LAZ layers activate both catabolic and anabolic wall modification pathways during the abscission program to facilitate organ shedding and to develop protective cell layers. The data also indicate that some transcripts preferentially expressed in LAZ and associated with protein biosynthesis and modification, (ubiquitination and phosphorylation) might play specific and relevant roles in ethylene-promoted citrus leaf abscission. On the other hand, the results highlight the activation of defensive programs in the contiguous Pet. In addition, new potential citrus abscission-regulatory genes were identified. The involvement of particular members of different transcription factor gene families (MADS-box, basic helix-loop-helix and homeodomain proteins) in the differentiation of the effective cell separation layer is also suggested.

In conclusion, the combined LCM and microarray hybridization approach applied in this survey, exemplified by the study of ethylene-promoted citrus leaf abscission, proved to be a powerful tool for elucidating the genetic regulation of cell-specific processes. In addition, this work provides many potential candidate genes for further biotechnological approaches and modifications, especially transcription factors and genes involved in signaling events.

## Methods

### Plant material

Clementine mandarin (*Citrus clementina *Hort. Ex Tan. cv. Clemenules) mature hardened leaves were collected from adult trees grown in a homogeneous experimental orchard under normal cultural practices at the Instituto Valenciano de Investigaciones Agrarias. The leaf explants, prepared as previously described [18], were incubated for either 24 or 48 h in the presence or absence of ethylene (10 μl/l).

### Cryoscanning electron microscopy (cryo-SEM)

To examine the proximal (petiole) and distal (leaf blade) fracture plane of the ethylene-activated LAZ by cryo-SEM, the petiole of leaf explants was forcibly separated from the leaf blade. Both portions were mounted on SEM stubs attached to the specimen holder of a CT-1000C Cryo-transfer system (Oxford Instruments) and immediately frozen in nitrogen slush (-210°C). The frozen mounted specimens were first transferred to the cryo-stage of a JEOL JSM-5410 scanning electron microscope and after that to the sample-stage where the condensed surface water was sublimed by controlled warming to -85°C. The mounted specimens were transferred again to the cryo-stage and sputter coated with a thin film of gold. At least three pairs of separated leaf explants, non-treated and treated with ethylene for either 24 or 48 h, were observed at an accelerating voltage of 10 keV at 50× and 500× magnification.

### Preparation of laminar abscission zone-containing tissue, sectioning and laser capture microdissection (LCM)

Four mm wide portions of LAZ-containing leaf tissue were dissected from 24 h ethylene-treated leaf explants and immediately snap-frozen in OCT embedding medium (Labonord Cryoblock, France) in Peel-A-Way disposable plastic tissue embedding moulds (Polysciences Inc., Warrington, PA, USA). The embedded samples were stored at -80°C until used. Four LAZ-containing leaf portions were arranged in each embedding mould. Longitudinal sections 10 μm thick were cut with a Leica CM1900 cryostat (Leica Microsystems, Germany) at -20°C. Cryosections were mounted on PET-membrane-coated stainless steel slides (Leica Microsystems, Germany) and kept twice for 10 min in 70% ethanol at -20°C followed by three xylene steps for 10 min at room temperature in a fume hood. Slides were air-dried and immediately microdissected. A Leica AS Laser Microdissection system (Leica Microsystems, Inc., Germany) was used for isolation of cells from prepared tissue sections. Cells from the LAZ and the adjacent Pet were selected for LCM from 25-30 cryosections. Dissection was performed with the following settings: 10× magnification, aperture: 15, intensity: 45, speed: 3, bridge: medium and offset: 22. Microdissected tissue was collected in the cap of a 0.5 ml microtube. LAZ and Pet were collected separately. To avoid sample heating by the microscope lamp, the collection time for each sample was less than 1 h.

### RNA isolation, sample labelling and microarray hybridization

Two independent biological replicates were collected for each cell type. For each independent sample, total RNA from ~15,000 pooled cells was extracted using the RNeasy Micro Kit (Qiagen) following the manufacturer's instructions. This kit is specially recommended for Leica microdissected tissue in order to keep integrity. The RNA yield was typically 1.7-1.8 ng/μl. The RNA purity was assessed by measurements of OD_260_/OD_280_. Two RNA amplification rounds were performed utilizing the TargetAmp™ 2-Round Aminoallyl-aRNA Amplification Kit (EPICENTRE Biotechnologies, Madison, WI, USA) following the manufacturer's instructions. The quality of the amplified RNA was evaluated by OD_260_/OD_280 _measurements and agarose gel electrophoresis (see Figure 2). Each sample was labelled once with Cy3 labelling and once with Cy5, ensuring a dye balance, and a previously described cDNA citrus microarray was utilized [23]. Hybridized arrays were scanned with a Scanarray Gx scanner (PerkinElmer) equipped with the Scanarray Express software to obtain an appropriate photomultiplier gain ratio for the two channels and a percentage of 1% of saturated spots. We used the GenePix 4.1 software (Axon Instruments; compatible with Scanarray Express) to transform the intensity into numeric data and for data acquisition. Spots flagged as 'not found' or 'bad' during the scanning, and those displaying a signal-to-background ratio <2 were discarded. The Lowess method was used for Normalization. Probes showing significant differential gene expression were identified using the Linear Models in Microarrays (LIMMA) library [93] of the Bioconductor software package [94]. Gene expression differences were only considered significant under a P-value lower than 0.05 and an M contrast cutoff value of +/- 1, being M = log2 [LAZ/Pet]. Positive or negative probe values corresponded to genes preferentially expressed in LAZ or Pet, respectively. The raw microarray data as well as the protocols used to produce the data and the normalized data were deposited in the ArrayExpress database under the accession number E-MEXP-1428. Functional classification of the selected genes was performed using MIPS (Munich Information Center for Protein Sequences, ) categorization. Amplified RNA from the same samples described above was used for PCR. Microarray hybridization data were confirmed by semi-quantitative RT-PCR and real-time RT-PCR analysis (see Figures 5 and 11).

### Semi-quantitative RT-PCR and real-time RT-PCR

RNA concentration values were determined by performing three fluorometric assays per RNA sample using RiboGreen dye (Molecular Probes) following the manufacturer's instructions. Quantitative one-step real-time RT-PCR was performed with a LightCycler 2.0 Instrument (Roche) equipped with Light-Cycler Software 4.0, as previously described [95]. Transformation of fluorescence intensity data into relative mRNA levels was performed using a 10-fold dilution series standard curve of a single RNA sample. Relative mRNA levels were normalized to total RNA amounts as previously described [96,97]. Specificity of the amplification reactions was assessed by PCR-product sequencing and by post-amplification dissociation curves. Primer sequences and the sizes of the fragments are listed in the Supplementary Information (Additional File 8).

Semi-quantitative RT-PCR analysis was carried out using the SuperScript II Reverse Transcriptase kit from Invitrogen (Carlsbad, CA 92008, USA) according to the manufacturer's instructions. First-strand cDNA synthesis was performed in a 20 μl reaction containing 500 ng of mRNA, 1 μl of 500 μg/ml oligo (dT), 1 μl of 10 mM dNTPs, 4 μl of 5× First-Strand Buffer, 2 μl of 0.1 M DTT, 1 μl of RNaseOUT (Invitrogen), 1 μl of SuperScript II RT and 12 μl of water.

PCR reactions were performed in 50 μl using the Biotools *Taq *DNA Polymerase (BIOTOOLS, B&M Labs, S.A., Spain); the PCR conditions for amplification were: 5 min at 95°C, 28 to 35 cycles of 95°C for 30 s, 55 to 57°C (depending on the primer combination) for 30 s, 72°C for 30 s, and 7 min at 72°C, 4°C. PCR products were run on a 1% agarose gel to check the size and the intensity of the expected band. Citrus actin-binding protein (CitABP) was used as a reference to evaluate the amounts of mRNA in both LAZ and Pet.

## Abbreviations

AZ: abscission zone; EST: expressed sequence tag; LAZ: laminar abscission zone; LCM: laser capture microdissection; LTP: lipid transfer protein; Pet: petiolar cortical; PI: proteinase inhibitor; PR: pathogenesis-related protein; RING: RING-finger domain protein; RLK: receptor-like kinase; ROS: reactive oxygen species; UPS: ubiquitin/proteasome system.

## Authors' contributions

JA initiated the study, produced the ethylene-induced LAZ and Pet samples for further analyses, carried out the fresh tissue embedding sample preparation, the cryosections, laser microdissection, RNA extraction, RNA amplification and microarray hybridizations, performed the real-time PCR and contributed to the morphological characterization of ethylene-induced LAZ and to the study design. PM carried out the semiquantitative RT-PCRs and the real-time PCR, and participated in the plant material sample handling and the array analyses. MC performed the data normalization and microarray expression comparison and contributed to the array analyses. FRT performed the morphological characterization of ethylene-induced LAZ, coordinated and performed the data analysis and interpretation, designed the fresh tissue embedding and LCM strategies, drafted the manuscript and produced the study design. MT's contribution was crucial for the study design, data interpretation and the final manuscript version. All authors contributed to the final version of the manuscript. All authors read and approved the final manuscript.

## Supplementary Material

Additional file 1 **List of citrus ESTs associated to cell-wall remodeling**. Expressed sequence tags (ESTs) and the corresponding genes associated with cell wall remodeling expressed preferentially in the laminar abscission zone cells (M>1) or in the petiolar cortical cells (M<1). Click here for file

Additional file 2 **List of citrus ESTs associated to cell wall biosynthesis**. Expressed sequence tags (ESTs) and the corresponding genes associated with cells wall biosynthesis expressed preferentially in the laminar abscission zone cells (M>1) or in the petiolar cortical cells (M<1). Click here for file

Additional file 3 **Overview of metabolic steps and compartmentalization of enzymes involved in cell wall biosynthesis**. Positive values of the gene expression ratio (log2 [LAZ/Pet]) show transcripts involved in pyruvate metabolism, glycolysis or nucleotide-sugar interconversions preferentially expressed in LAZ and negative values those preferentially expressed in Pet. Each bar represents the expression ratio of a singleton or of different ESTs assembled in the same contig. Data are the average of two dye-swap comparisons and error bars show SE. Click here for file

Additional file 4 **List od citrus ESTs associated to protein biosynthesis and metabolism**. Expressed sequence tags (ESTs) and the corresponding genes associated with protein biosynthesis and metabolism expressed preferentially in the laminar abscission zone cells (M>1) or in the petiolar cortical cells (M<1). Click here for file

Additional file 5 **List of citrus ESTs associated with defense and interaction with the environment**. Expressed sequence tags (ESTs) and the corresponding genes associated with defense and interaction with the environment expressed preferentially in the laminar abscission zone cells (M>1) or in the petiolar cortical cells (M<1). Click here for file

Additional file 6 **List of citrus ESTs associated with protein phosphorylation and signal transduction**. Expressed sequence tags (ESTs) and the corresponding genes associated with protein phosphorylation and signal transduction expressed preferentially in the laminar abscission zone cells (M>1) or in the petiolar cortical cells (M<1). Click here for file

Additional file 7 **List of citrus ESTs asociated with transcriptional regulation**. Expressed sequence tags (ESTs) and the corresponding genes associated with transcriptional regulation expressed preferentially in the laminar abscission zone cells (M>1) or in the petiolar cortical cells (M<1). Click here for file

Additional file 8 Genes tested with sqRT-PCR and qRT-PCR and primers used. Click here for file
