# Supplementary figures and images for: Comparative transcriptional survey between laser-microdissected cells from laminar abscission zone and petiolar cortical tissue during ethylene-promoted abscission in citrus leaves
Source: BMC Plant Biol. 2009 Oct 23;9:127. doi: 10.1186/1471-2229-9-127 (PMC2770498; doi:10.1186/1471-2229-9-127)

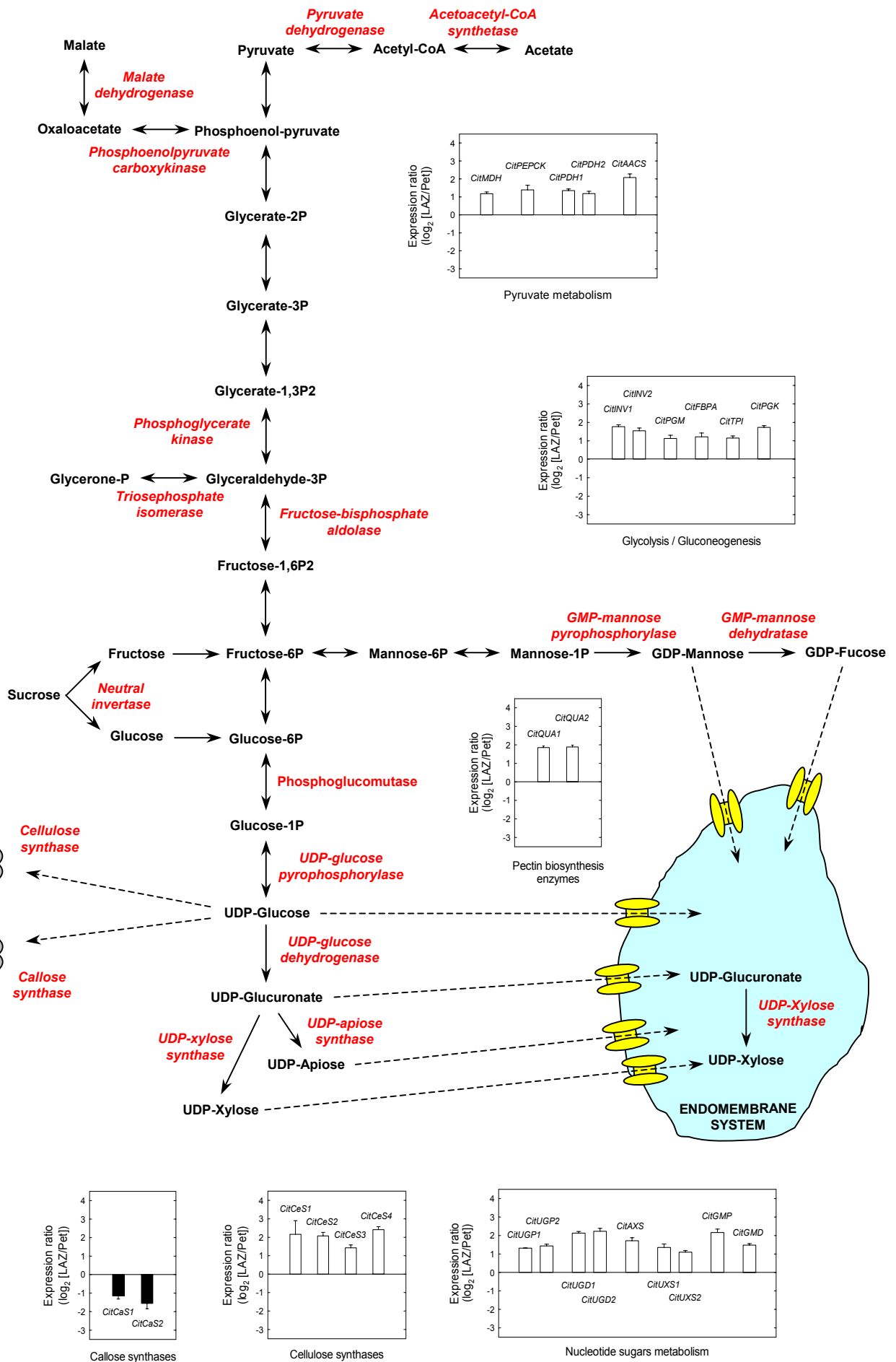

Supplement: Additional file 3 — Overview of metabolic steps and compartmentalization of enzymes involved in cell wall biosynthesis. Positive values of the gene expression ratio (log2 [LAZ/Pet]) show transcripts involved in pyruvate metabolism, glycolysis or nucleotide-sugar interconversions preferentially expressed in LAZ and negative values those preferentially expressed in Pet. Each bar represents the expression ratio of a singleton or of different ESTs assembled in the same contig. Data are the average of two dye-swap comparisons and error bars show SE. [file 1471-2229-9-127-S3.PDF]
